# Mechanisms and Materials for NTE

**DOI:** 10.3389/fchem.2018.00371

**Published:** 2018-08-22

**Authors:** J. Paul Attfield

**Affiliations:** Centre for Science at Extreme Conditions and School of Chemistry, University of Edinburgh, Edinburgh, United Kingdom

**Keywords:** thermal expansion, negative thermal expansion, thermal expansion coefficient, structural NTE, electronic NTE, morphological NTE

## Abstract

Negative thermal expansion (NTE) upon heating is an unusual property but is observed in many materials over varying ranges of temperature. A brief review of mechanisms for NTE and prominent materials will be presented here. Broadly there are two basic mechanisms for intrinsic NTE within a homogenous solid; structural and electronic. Structural NTE is driven by transverse vibrational motion in insulating framework–type materials e.g., ZrW_2_O_8_ and ScF_3_. Electronic NTE results from thermal changes in electronic structure or magnetism and is often associated with phase transitions. A classic example is the Invar alloy, Fe_0.64_Ni_0.36_, but many exotic mechanisms have been discovered more recently such as colossal NTE driven by Bi–Ni charge transfer in the perovskite BiNiO_3_. In addition there are several types of NTE that result from specific sample morphologies. Several simple materials, e.g., Au, CuO, are reported to show NTE as nanoparticles but not in the bulk. Microstructural enhancements of NTE can be achieved in ceramics of materials with anisotropic thermal expansion such as beta–eucryptite and Ca_2_RuO_4_, and artificial NTE metamaterials can be fabricated from engineered structures of normal (positive) thermal expansion substances.

## Introduction

NTE (negative thermal expansion) refers to the unusual phenomenon of volume contraction upon heating. Although most materials display positive thermal expansion (PTE) on heating, NTE is found in a wide variety of substances over varying ranges of temperature. This brief review is an attempt to summarize the mechanisms and prominent materials that show NTE. Further details may be found in more substantial reviews published by other authors in recent years (Lind, 2012; Takenaka, 2012; Chen et al., 2015; Dove and Fang, 2016; Liu et al., 2018; Mittal et al., 2018) and in the other papers in this special issue.

Control of thermal expansion is important for many applications from ceramic cooker hobs to housings for optical devices, with zero thermal expansion (ZTE) materials or composites of PTE and NTE components being particularly useful. Thermal expansion is quantified through the linear or volume (bulk) thermal expansion coefficients (TECs), α_*L*_ = (1/*L*)(d*L*/d*T*), and α_*V*_ = (1/*V*)(d*V*/d*T*), which respectively measure the change in length *L* or volume *V* of an object with temperature *T*. α_*L*_ and α_*V*_ are typically quoted in 10^−6^ K^−1^ units, equivalent to ppm (parts per million) K^−1^ or MK^−1^. Isotropic substances such as simple liquids, glasses, polycrystalline ceramics, and cubic crystals, have the same α_*L*_ in all directions with α_*V*_ = 3α_*L*_. ZrW_2_O_8_ is a famous example of a cubic NTE material (Mary et al., 1996). However uniaxial (tetragonal, hexagonal, or trigonal) crystals may have different linear TECs α_||_ and α_⊥_ parallel and perpendicular to the unique symmetry axis, respectively, and crystals with orthorhombic or lower symmetry have three different values α_1_, α_2_, and α_3_ in mutually perpendicular directions. The volume TEC is given by α_*V*_ = α_||_ + 2α_⊥_ or α_*V*_ = α_1_ + α_2_ + α_3_ and when the linear TECs are very different, such as a mix of negative and positive values, then highly anisotropic thermal expansion may be obtained. TECs of crystalline materials are usually measured by determining how unit cell lengths change with temperature from diffraction measurements. Direct strain gauge (dilatometry) measurements of crystals can also be used, and are particularly useful for ceramics and amorphous materials such as glasses. Diffraction and dilatometry expansion measurements can give different results due to the effects of microstructure, as discussed in section Microstructural NTE.

NTE materials have negative α_*V*_ over some temperature range. Reported α_*V*_ values vary from −1 to −1,000 × 10^−6^ K^−1^, but it is also important to consider the temperature range to which the quoted α_*V*_ refers as very large negative α_*V*_'s may result from modest volume decreases over very narrow temperature ranges at a phase transition. For this reason it is often more useful to consider the overall volume decrease; materials with –Δ*L*/*L* > 1% and so –Δ*V*/*V* > 3% have notably large NTE.

The thermodynamic origin of thermal expansion in solids is expressed through the relation α_*V*_ = γ*C*_*V*_/*BV* where *C*_*V*_ is heat capacity at constant *V, B* = –*V*(d*p*/d*V*) is the bulk modulus with *p* being pressure, and γ is the weighted or macroscopic Grüneisen parameter summed over values for the active phonon frequencies ω_*i*_ as γ_*i*_ = –d(ln ω_*i*_)/d(ln *V*). *C*_*V*_ and *B* always take positive values, hence variations in the sign of α_*V*_ arise from corresponding variations in the sign of γ. NTE is often associated with other unusual lattice properties such as negative linear compressibility under applied pressure (Mittal et al., 2018) and pressure-induced softening where d*B*/d*p* becomes negative (Dove and Fang, 2016).

**Figure 1:**
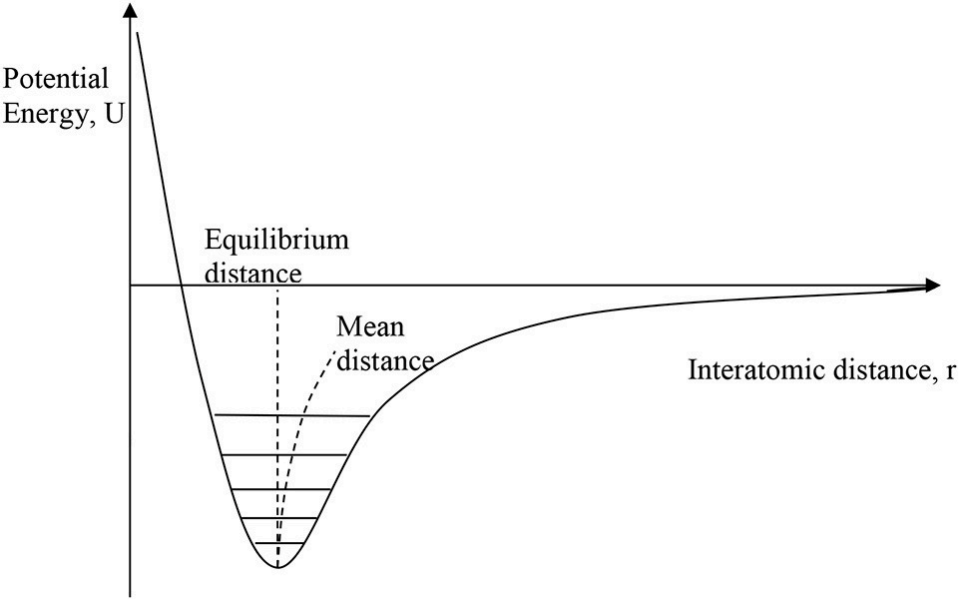
The interatomic bonding potential, showing the variation of potential energy with interatomic distance for a pair of bonded atoms. Vibrational energy levels are quantised and with increasing temperature the occupation of higher levels leads to a slight increase in the mean interatomic distance relative to the minimum–energy equilibrium distance as shown.

Conventional PTE arises because γ is usually positive as a consequence of the shape of the interatomic potential for bonding between two atoms, as shown in Figure 1. Anharmonicity in the shape of the potential leads to an increase in the average interatomic distance as higher vibrational states become more populated as temperature rises. As this pairwise potential shape applies qualitatively to all type of chemical bonding, it might appear that PTE should be a universal behavior, but NTE can arise from two “escapes” that circumvent the latter argument.

## Intrinsic NTE mechanisms

### Structural NTE

**Figure 2:**
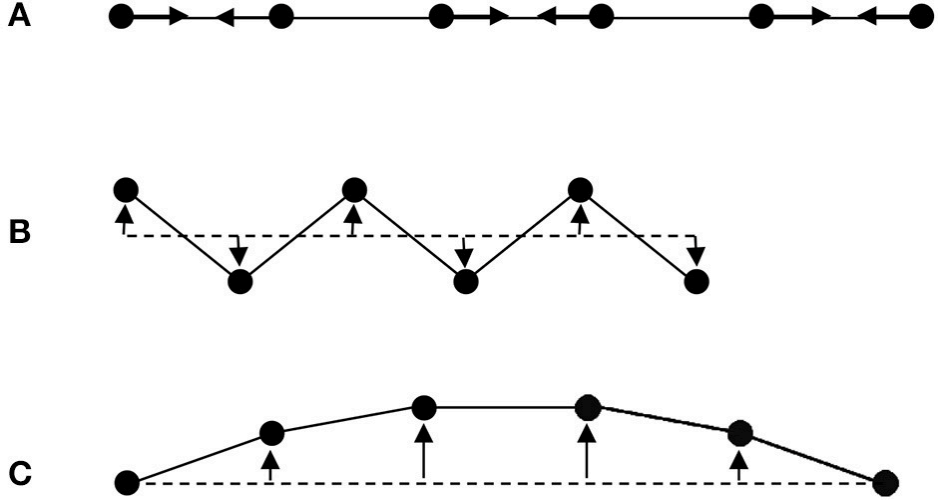
Schematic illustrations of lattice modes for a chain of atoms. **(A)** Longitudinal optic (LO) mode leading to PTE through asymmetry of the pairwise bonding potential shown in Figure 1. **(B)** High energy transverse optic (TO) mode that greatly shortens the chain giving large NTE as the vibrational amplitude increases with temperature. **(C)** Low energy transverse acoustic (TA) mode leading to more modest NTE as temperature increases.

The first “escape” from the universal PTE behavior expected from the interatomic potential in Figure 1 arises from the more complex vibrational properties of large arrays of atoms. Figure 2 shows some of the possible motions for a chain of atoms. Longitudinal (L) vibrations in the direction of the bonds (Figure 2A) tend to lengthen the chain as temperature increases through thermal expansion of the individual bonds via the anharmonicity of the interatomic potential (Figure 1). However, transverse (T) motions perpendicular to the direction of the chain tend to shorten the chain–length as the amplitude of vibration increases with temperature (Figures 2B,C) and so can lead to NTE. This is sometimes known as the tension or “guitar string” effect. Lattice vibrations, also known as phonons, are usefully classified as optic (O) with short wavelengths and high frequencies and energies, or acoustic (A) with long wavelengths and low frequencies and energies. Transverse optic (TO) phonons like that shown in Figure 2B lead to large chain shortening (NTE) but may only be excited at high temperatures in view of their high energies, whereas transverse acoustic (TA) modes that lead to more modest NTE are excited at lower temperatures. Detailed theoretical and experimental analyses of phonon spectra are needed to assess the contributions of TO and TA vibrations to the NTE of real materials (Dove and Fang, 2016; Mittal et al., 2018).

Structural NTE results when the shortening effects of the transverse phonon amplitudes due to bending or torsional motions outweigh the expansion effects of the longitudinal modes. Low atomic connectivity so that atoms have free space to move into during transverse motions leading to large amplitudes is a necessary feature for NTE to prevail. Planar 3-coordination is the maximum connectivity known to lead to negative expansion, as exemplified by graphene sheets of carbon atoms (Yoon et al., 2011). However when these sheets are stacked in the three-dimensional lattice of graphite, conventional PTE arising from soft van der Waals bonding potentials in the stacking (high symmetry) direction gives a large α_||_ = 23.1 × 10^−6^ K^−1^ that outweighs the NTE in the perpendicular directions (α_⊥_ = −0.6 × 10^−6^ K^−1^) leading to bulk PTE (α_V_ = α_||_ + 2α_⊥_ = 21.9 × 10^−6^ K^−1^) (Morgan, 1972).

Bulk structural NTE requires a large proportion of 2-coordinate linker groups, connecting more highly-coordinated atoms into a three-dimensional structure. Some representative examples of structural NTE material types, with 2-connected linkers underlined, are ScF_3_, Ag_2_O, ZrW_2_O_8_, ZrV_2_O_7_, LiAlSiO_4_ (the mineral β-eucryptite), zeolitic forms of SiO_2_ (e.g., ITQ-4) and related AlPO_4_ (e.g., AlPO_4_-17) frameworks, Cd(CN)_2_, Co_3_[Co(CN)_6_]_2_, and metal organic frameworks such as IRMOF-1 (Zn_4_O(bdc)_3_, where bdc is 1,4-benzodicarboxylate). These have α_*V*_ values of magnitude −20 to −120 × 10^−6^ K^−1^ over typical temperature ranges of a few hundred K; details and citations are shown in Dove and Fang (2016). All of these materials have the majority of their atoms in the 2-connected linkers. The metal fluoride and oxide examples have 2-coordinate atoms linking tetrahedral or octahedral units together. These polyhedra tend to be rigid so the transverse vibrations of the lattice may be described in the rigid unit mode (RUM) picture (Dove and Fang, 2016).

The importance of the transverse motions of the linker atoms or groups to NTE is further demonstrated by changes observed when additional molecules or ions are inserted into NTE materials. The inserted species within cavities in the structure are adjacent to the linkers and so reduce the amplitudes of their transverse vibrations. Redox insertion of only 6% Li into Fe-doped ScF_3_ switches the TEC from negative to positive (Chen et al., 2017), and the same change is found when K^+^ or H_2_O is inserted into the channels of the cyanide framework material YFe(CN)_6_ (Gao et al., 2017).

### Electronic NTE

**Figure 3:**
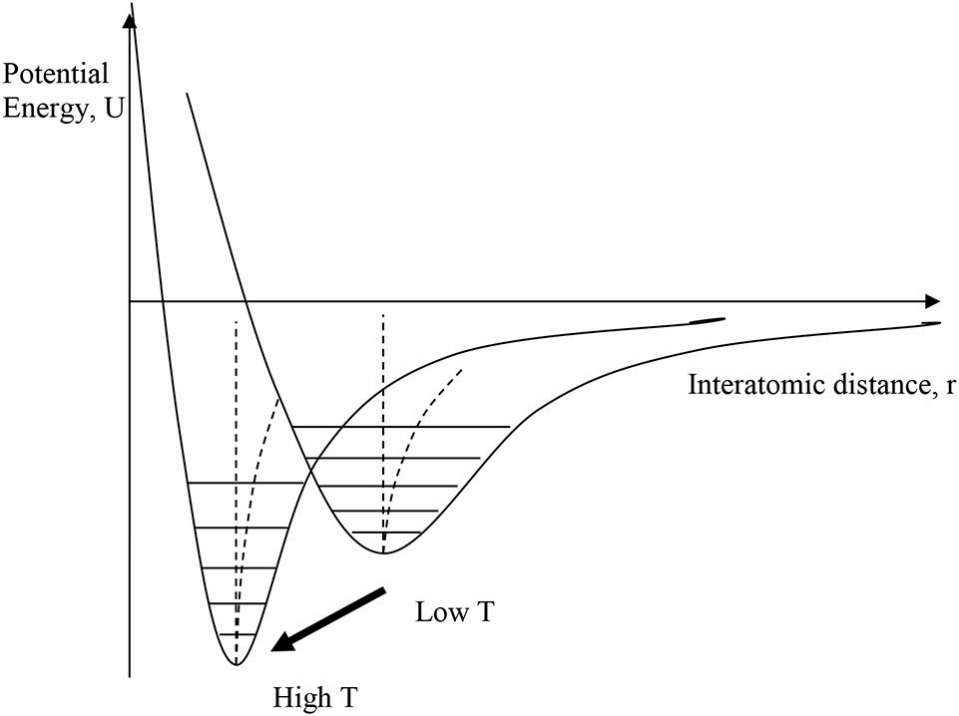
Illustration of electronic mechanisms for NTE, where the interatomic bonding potential changes with temperature, moving to a more strongly bonding curve with smaller equilibrium distance at higher temperature. This may occur through a relatively sharp first order phase transition between two distinct states, or the potential may gradually evolve with changing temperature.

A disparate group of materials, usually dense metal alloys or ceramics, display NTE that is not driven by the structural (transverse vibration) mechanism. Although they have a wide variety of physical properties, and so are sometimes described as having different NTE mechanisms, they have the common feature that NTE results from thermal changes in the interatomic bonding potential, as illustrated in Figure 3. Changes in bonding with temperature such that the interatomic potential becomes more strongly bonding can shift the curve to a smaller equilibrium distance at higher temperature. This may occur through a relatively sharp first order phase transition between two distinct states, or the potential may gradually evolve with changing temperature through a second or higher order transition. When the effect of this “escape” outweighs the usual PTE from the anharmonicity of the potentials then NTE may be observed over temperature range of the crossover.

**Figure 4:**
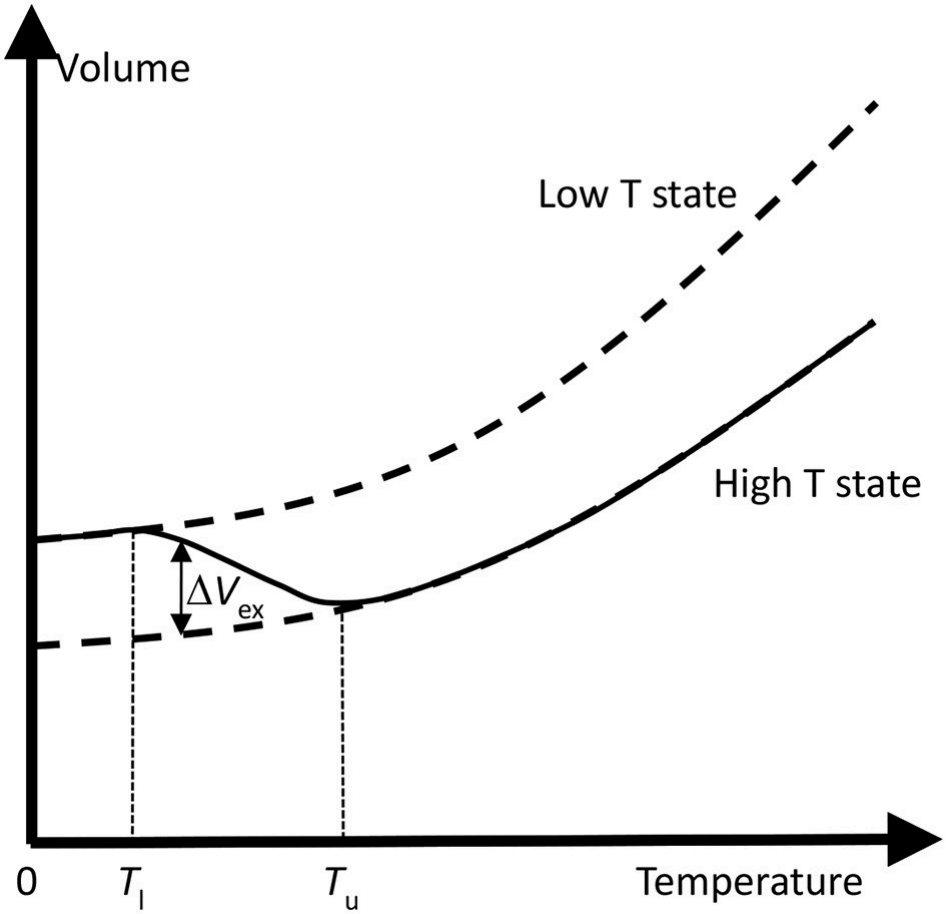
Schematic changes in lattice volume with temperature for a material displaying electronic NTE. The change from the larger-volume low-*T* to the smaller-volume high-*T* states (as shown in Figure 3) leads to NTE in the crossover region with lower and upper temperatures *T*_l_ and *T*_u_. *T*_u_ often marks an ordering temperature such as a magnetic or ferroelectric Curie transition. The electronically-induced excess volume Δ*V*_ex_ relative to the extrapolated volume of the high-*T* state is shown.

Figure 4 illustrates the schematic changes in lattice volume with temperature for a material displaying electronic NTE. Both the larger-volume low-*T* and the smaller-volume high-*T* states shown in Figure 3 display conventional PTE behavior, but the change between them in the crossover region with lower and upper temperatures *T*_l_ and *T*_u_ leads to NTE. *T*_u_ often marks an ordering temperature such as a magnetic or ferroelectric Curie transition, and the lower limit *T*_l_ is reached where the order parameter (magnetization or electric polarization) is fully saturated. In other cases such as charge transfer materials, *T*_l_ and *T*_u_ mark the lower and upper limits of the two-phase region where the low-*T* and high-*T* phases coexist. The electronically-induced excess volume Δ*V*_ex_ relative to the extrapolated volume of the high-*T* state, *V*_HT_, may be used to calculate the spontaneous volume striction Δ*V*_ex_/*V*_HT_. Variations in the magnitude of Δ*V*_ex_ and in the separation between *T*_l_ and *T*_u_ may be used to tune electronic materials from reduced PTE, through ZTE to NTE behavior. A famous material that launched the study of unusual thermal expansion properties is the Invar alloy Fe_0.64_Ni_0.36_ named for an *invariant* length with a very small α_*L*_ = 1 × 10^−6^ K^−1^ (effectively ZTE) below magnetic *T*_C_ = 500 K.

The transitions or changes that give rise to electronic NTE are usually associated with an increase in electron delocalization on passing from the low-*T* to the high-*T* phase. The low-*T* phase is more electron localized or correlated (e.g., magnetically ordered), due to electron-electron repulsions that may be described by the Hubbard *U* energy, while the more electron delocalized or disordered high-*T* phase is stabilized by entropy. Electrons near the Fermi level are often in d- or f-states that are non-bonding or weakly antibonding, so their delocalization allows the bonding potential to become more negative and shift to shorter distance as shown in Figure 3.

Classes of materials where electronic NTE is found are;

- Metallic magnets, notably the Invar alloy above, also R_2_Fe_17_ (R = rare earth) and related intermetallics, the permanent magnet family R_2_Fe_14_B, the Mn_3_(Cu,Ge)N antiperovskites, and metallic perovskite oxides SrRuO_3_ and manganites such as La_0.8_Ba_0.2_MnO_3_. NTE is usually observed over a wide temperature range below the magnetic Curie transition.
- Insulating magnets—these generally do not show NTE but some examples are found for frustrated spinels e.g., CdCr_2_O_4_ and CdCr_2_S_4_, the multiferroic Pb(Fe_0.5_Nb_0.5_)O_3_, and orbitally ordered MnF_3_, below their Curie or Néel temperatures.
- Charge transfer materials, e.g., Bi_0.95_La_0.05_NiO_3_, LaCu_3_Fe_4_O_12_, Sm_0.67_Y_0.33_S, Sm_2.75_C_60_, and Yb_8_Ge_3_Sb_5_, can show very large NTE resulting from volume collapses at metal to insulator charge transfer transitions. Bi_0.95_La_0.05_NiO_3_ has α_*V*_ = −410 × 10^−6^ K^−1^ between 300 and 370 K, termed colossal NTE (CNTE). The oxides have intermetallic Bi/Ni or Cu/Fe charge transfer transitions, whereas electrons released through the ionization process R^2+^ → R^3+^ + e^−^ in the R = Sm, Yb materials are delocalized in the conduction band at high temperatures. NTE in V_2_OPO_4_ where charge ordering occurs without a metal-insulator transition was recently reported (Pachoud et al., 2018).
- Orbital ordering transitions usually associated with first-order Jahn-Teller distortions give rise to NTE in several materials. Ca_2_RuO_4_ has orbital order associated with a metal-insulator transition, while insulating LaMnO_3_ which is much studied as the parent phase of the perovskite manganites undergoes a 0.4% cell volume decrease at 750 K where the orbital order-disorder transition occurs.
- Ferroelectrics associated with off-center displacements of cations to give a net polarization sometimes show NTE below their Curie transitions. Prominent examples are perovskite oxides of lead with transition metal cations showing a second-order Jahn-Teller effect such as PbTiO_3_, Pb(Mg_0.33_Ta_0.67_)O_3_, and Pb(Fe_0.5_Nb_0.5_)O_3_. Cooling below *T*_C_ leads to a change from symmetric O–M–O–M–O to asymmetric O^……^M-O^……^M-O chains of atoms leading to polarity in the chain direction. The lengthening of O^……^M bonds where electrons are localized in antibonding states outweighs the shortening of the M-O bonds, leading to a net expansion in the polar chain direction on cooling and hence the excess volume associated with NTE below *T*_C_. This can lead to very large volume collapses on warming, for example, Pb_0.8_Bi_0.2_VO_3_ shows Δ*V*/*V* = −7.9% around *T*_C_ ≈ 600 K (Yamamoto et al., 2018).
- Superconductors sometimes show an excess volume and NTE below their critical temperatures *T*_c_, for example in MgB_2_, La_1.85_Sr_0.15_CuO_4_, and NdFeAsO_0.89_F_0.11_. Electron-phonon coupling is directly implicated in the superconducting mechanism for BCS-type MgB_2_, whereas the latter two materials are unconventional superconductors where a magnetic pairing mechanism may be important. Loss of bonding electron density at the Fermi level due to electron-pairing below *T*_c_ is the general cause of NTE in superconductors.

Further details and citations when not shown above are given in the comprehensive review of NTE in functional materials by Chen et al. (2015).

## Morphological NTE

The structural and electronic mechanisms for NTE described above apply to chemically homogenous materials such as a single crystal. However, there are further instances where NTE can arise or differ from the normal bulk behavior due to the specific morphology of the sample.

### Nanoparticle NTE

A variety of materials that show bulk PTE have been found to display NTE when prepared as small particles, usually in the nanoscale regime. For example, the magnetic insulators CuO and MnF_2_ show PTE in the bulk but as 5 nm particles they display NTE below their Néel temperatures. CuO has a giant NTE of α_*V*_ = −110 × 10^−6^ K^−1^ between 20 and 170 K (Zheng et al., 2008). Nanoparticles of Au (Li et al., 2002) and TiO_2_ (Zhu et al., 2016) are also reported to display NTE.

The origin of nanoparticle NTE is usually electronic as, for example, the excess volume of CuO particles above follows the general behavior shown in Figure 4. Localization of ordered or correlated states tends to be enhanced at and near surfaces so the associated lattice expansion on cooling can dominate the overall behavior in small particles with a large proportion of surface atoms. The structural NTE mechanism could also play a part as surface atoms have a lower connectivity than the bulk so transverse vibrational amplitudes may be enhanced.

### Microstructural NTE

**Figure 5:**
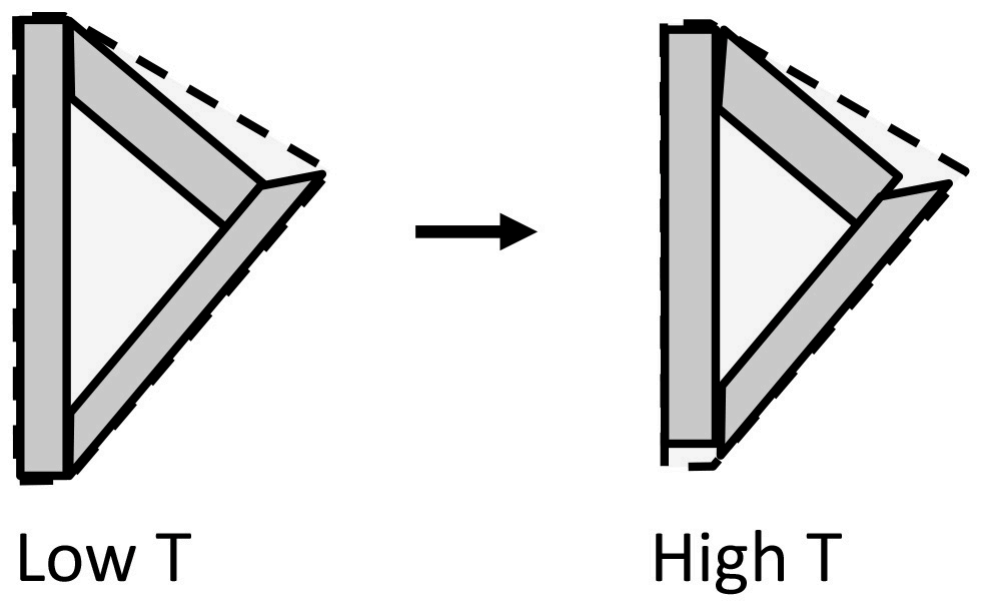
Illustrative two-dimensional model for the microstructural NTE effect, showing three crystallites enclosing a void. The crystallites shown have contracted by 10% along their long axis and expanded by 10% in the perpendicular direction on changing from low *T* to high *T*, so their total area has not changed. However, the enclosing area for the ensemble shown by broken lines undergoes an overall decrease of 8%, illustrating how anisotropic thermal expansion may lead to overall NTE.

Direct measurement of α_*V*_ for ceramic samples sometimes gives a more negative value than that expected from crystallographic measurement of the linear TECs. The excess negative expansion is achieved by reducing the volumes of microcracks or voids within the ceramic on heating, as illustrated schematically in Figure 5. Microstructural NTE was reported in an early study of β-eucryptite (LiAlSiO_4_) (Gillery and Bush, 1959) and very large effects have recently been discovered in Ca_2_RuO_4_ (Takenaka et al., 2017a). These ceramic materials both have anisotropic thermal expansion with one strongly negative coefficient that dominates the expansion of their ceramics. For example, on heating from 150 to 340 K, the orthorhombic *a, b*, and *c* axes of Ca_2_RuO_4_ show length changes of −0.6, −5.0, and +4.5% respectively, so an overall Δ*V*/*V* = −1.1% is expected. However an 80% dense ceramic sample gave Δ*V*/*V* = −6.7% revealing a substantial microstructural NTE effect.

Anisotropic thermal expansion leads to stresses at grain boundaries that result in microcracking in β-eucryptite ceramics on cooling to room temperature after sintering. Reduction of the microcrack volumes on subsequent heating results in bulk NTE and an enhancement of the excess NTE with increasing grain size (and hence internal microcrack volume) has been established (Pelletant et al., 2012). However, this is less problematic for Ca_2_RuO_4_-based ceramics, which have been combined with epoxy resin to generate ZTE materials that are stable to microcracking on thermal cycling (Takenaka et al., 2017b).

### Metamaterials NTE

**Figure 6:**
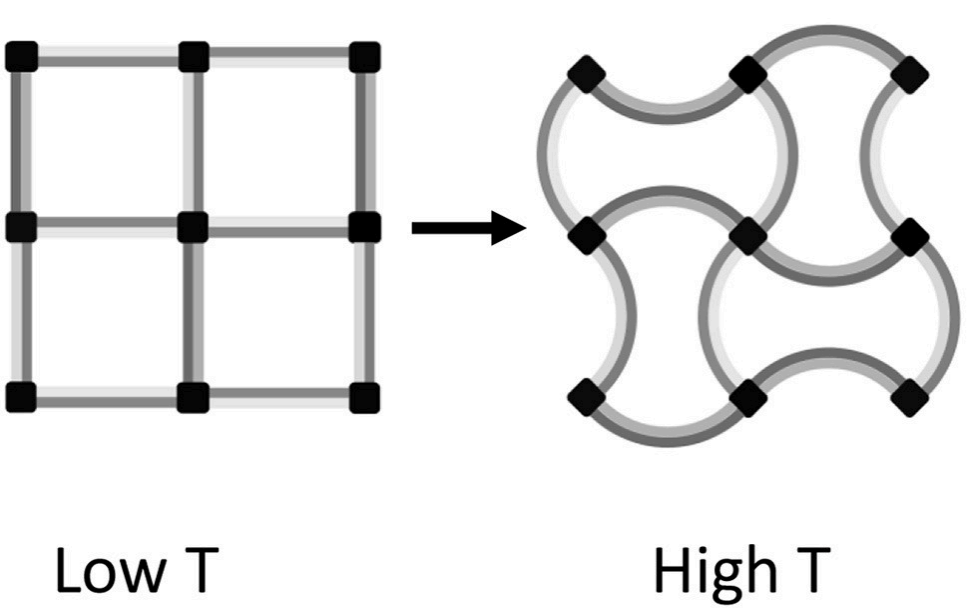
Illustrative two-dimensional example of an NTE metamaterial consisting of a square lattice of bimaterial strips acting as struts between 4-connected nodes. On heating from the low *T* to the high *T* state, the light and dark gray materials respectively expand in length by 6 and 17%, but the separation between nodes decreases by 4% corresponding to an 8% area contraction. Hence the metamaterial consisting of only PTE substances shows bulk NTE.

Artificial structures consisting of two or more materials with different TECs (which may all be positive) can be engineered to contract when heating. A simple example based on bonded strips of two materials with different positive TECs is shown in Figure 6. The structure contracts on heating through reduction of the volume of internal voids, which outweighs the increase in volume of the materials themselves. Designs for three-dimensional cellular metamaterials with varying TECs based on the use of such bimaterial connectors were proposed by Lakes (2007).

Recent developments in additive manufacturing through three-dimensional printing technologies have enabled metamaterials with bulk NTE to be generated. In a recent example, a two-component polymer metamaterial showed bulk negative expansion with α_*L*_ = −50 × 10^−6^ K^−1^ although the individual components had PTE with an average α_*L*_ = 40 × 10^−6^ K^−1^ (Qu et al., 2017).

## Summary

Although many diverse examples of NTE materials are reported, they may be classified according to two types of intrinsic mechanism. Electronic NTE arises from reduction in first-neighbor distances upon heating due to changes in the interatomic potential. These changes may arise from a variety of physical property transitions such as magnetism, charge transfer, ferroelectricity, and superconductivity that alter the distribution of electron density. Structural NTE occurs through reduction in second- or higher- neighbor distances upon heating due to dominant effects of transverse vibrations such as bending or torsional modes, and is found in many framework-type materials with a high proportion of 2-connected linkers.

The intrinsic NTE mechanisms may be enhanced in nanoparticles, most likely through electronic effects from localization of ordered or correlated states near surfaces. Further morphological mechanisms for NTE of bulk artifacts result from reducing the volume of internal voids upon heating. Microstructural NTE is found for ceramics of materials having anisotropic thermal expansion coefficients. Artificial structures of substances with different expansion coefficients may be used to generate metamaterials with NTE even when the individual components have PTE behavior.

## Author contributions

The author confirms being the sole contributor of this work and approved it for publication.

### Conflict of interest statement

The author declares that the research was conducted in the absence of any commercial or financial relationships that could be construed as a potential conflict of interest.
